# *Cryptosporidium parvum*-induced ileo-caecal adenocarcinoma and Wnt signaling in a mouse model

**DOI:** 10.1242/dmm.013292

**Published:** 2014-03-20

**Authors:** Sadia Benamrouz, Valerie Conseil, Magali Chabé, Marleen Praet, Christophe Audebert, Renaud Blervaque, Karine Guyot, Sophie Gazzola, Anthony Mouray, Thierry Chassat, Baptiste Delaire, Nathalie Goetinck, Nausicaa Gantois, Marwan Osman, Christian Slomianny, Vanessa Dehennaut, Tony Lefebvre, Eric Viscogliosi, Claude Cuvelier, Eduardo Dei-Cas, Colette Creusy, Gabriela Certad

**Affiliations:** 1 Ecologie et biodiversité, Faculté Libre des Sciences et Technologies de Lille, Université Catholique de Lille, Université Lille Nord de France, 59020 Lille, France.; 2 Biologie et Diversité des Pathogènes Eucaryotes Emergents (BDEEP), Centre d’Infection et d’Immunité de Lille (CIIL), Institut Pasteur de Lille, INSERM U1019, CNRS UMR 8402, Université Lille Nord de France, 59021 Lille, France.; 3 Faculté de Pharmacie, Université Lille Nord de France, 59021 Lille, France.; 4 Academic Department of Pathology, Ghent University, 9000 Ghent, Belgium.; 5 PEGASE-Biosciences, Institut Pasteur de Lille, F-59021 Lille, France.; 6 Gene Diffusion, 59501 Douai, France.; 7 Transcriptomic and Applied Genomic (TAG), Centre d’Infection et d’Immunité de Lille (CIIL), Institut Pasteur de Lille, INSERM U1019, CNRS UMR 8404, Université Lille Nord de France, 59021 Lille, France.; 8 Plateforme d’Expérimentations et de Hautes Technologies Animales, Institut Pasteur de Lille, 59021 Lille, France.; 9 Service d’Anatomie et de Cytologie Pathologiques, Groupe Hospitalier de l’Université Catholique de Lille, 59020 Lille, France.; 10 Centre Hospitalier Régional et Universitaire de Lille, Université Lille Nord de France, 59000 Lille, France.; 11 Centre AZM pour la Recherche en Biotechnologie et ses Applications, Laboratoire Microbiologie, Santé et Environnement, Université Libanaise, Tripoli, Lebanon.; 12 Inserm U1003, Laboratoire de Physiologie Cellulaire, Université Lille 1, 59655 Villeneuve d’Ascq CEDEX, France.; 13 Unité de Glycobiologie Structurale et Fonctionnelle, UMR CNRS 8576, IFR 147, Université Lille1, 59650 Villeneuve d’Ascq, France.

**Keywords:** SCID mouse model, Cryptosporidiosis, Wnt pathway, Cytoskeleton, Digestive cancer

## Abstract

*Cryptosporidium* species are apicomplexan protozoans that are found worldwide. These parasites constitute a large risk to human and animal health. They cause self-limited diarrhea in immunocompetent hosts and a life-threatening disease in immunocompromised hosts. Interestingly, *Cryptosporidium parvum* has been related to digestive carcinogenesis in humans. Consistent with a potential tumorigenic role of this parasite, in an original reproducible animal model of chronic cryptosporidiosis based on dexamethasone-treated or untreated adult SCID mice, we formerly reported that *C. parvum* (strains of animal and human origin) is able to induce digestive adenocarcinoma even in infections induced with very low inoculum. The aim of this study was to further characterize this animal model and to explore metabolic pathways potentially involved in the development of *C. parvum*-induced ileo-caecal oncogenesis. We searched for alterations in genes or proteins commonly involved in cell cycle, differentiation or cell migration, such as β-catenin, *Apc*, E-cadherin, *Kras* and *p53*. After infection of animals with *C. parvum* we demonstrated immunohistochemical abnormal localization of Wnt signaling pathway components and p53. Mutations in the selected loci of studied genes were not found after high-throughput sequencing. Furthermore, alterations in the ultrastructure of adherens junctions of the ileo-caecal neoplastic epithelia of *C. parvum*-infected mice were recorded using transmission electron microscopy. In conclusion, we found for the first time that the Wnt signaling pathway, and particularly the cytoskeleton network, seems to be pivotal for the development of the *C. parvum*-induced neoplastic process and cell migration of transformed cells. Furthermore, this model is a valuable tool in understanding the host-pathogen interactions associated with the intricate infection process of this parasite, which is able to modulate host cytoskeleton activities and several host-cell biological processes and remains a significant cause of infection worldwide.

## INTRODUCTION

*Cryptosporidium* species are apicomplexan parasitic protists that exist worldwide. The infection results from the ingestion of *Cryptosporidium* oocysts through the consumption of food or water that has been contaminated with fecal matter, or through direct person-to-person or animal-to-person contact (Chalmers and Katzer, 2013). This ubiquitous, intracellular parasite constitutes a large health risk to humans and animals. It causes self-limited diarrhea in immunocompetent persons and a life-threatening disease in immunocompromised persons (Ramirez et al., 2004). Contaminated water is the major source of *Cryptosporidium* infections for humans. Large-scale outbreaks of human cryptosporidiosis have been reported, often implicating contaminated drinking or recreational water as the cause (Ramirez et al., 2004; Rowan, 2011; Yoder and Beach, 2010). The ingestion of as few as ten oocysts can cause infection in immunocompetent persons (Okhuysen et al., 1999). This low infection threshold, together with the well-known resistance of *Cryptosporidium* oocysts to chlorine disinfection at concentrations typically applied in drinking-water plants, facilitates the waterborne transmission of cryptosporidiosis (Rowan, 2011; Yoder and Beach, 2010).

Nevertheless, key aspects of cryptosporidiosis remain unclear. For this reason, to contribute to the understanding of the dynamics of the infection, we previously developed an animal model of cryptosporidiosis that uses dexamethasone-treated or untreated adult severe combined immunodeficiency (SCID) mice infected orally with *Cryptosporidium parvum* or *C. muris* oocysts. Unexpectedly, we observed that SCID mice that had been infected with *C. parvum* developed digestive adenocarcinoma (Certad et al., 2007). Low- or high-grade intraepithelial neoplasia and invasive adenocarcinoma that is associated with numerous *C. parvum* life stages were detected in the digestive tract of SCID mice, including the stomach, ileo-caecal region and intrahepatic biliary tree (Certad et al., 2012; Certad et al., 2010a; Certad et al., 2010b; Certad et al., 2007). A highly significant correlation was found between the extension of cryptosporidiosis and the severity of neoplastic lesions (Certad et al., 2010b).

**TRANSLATIONAL IMPACT**

**Clinical issue**

*Cryptosporidium* coccidian parasites are a major cause of diarrhea worldwide. The infection results from ingestion of *Cryptosporidium* oocysts through the consumption of fecally contaminated food or water, or through direct contact. The infection causes self-limited watery diarrhea in immunocompetent individuals but has devastating effects in those who are immunocompromised. Despite its prevalence, the molecular mechanisms of cryptosporidiosis remain unclear. To explore the dynamics of this infection, an animal model of cryptosporidiosis using corticoid-treated or untreated adult SCID (severe combined immunodeficiency) mice, orally infected with *C. parvum* or *C. muris* oocysts, was previously developed by authors of this article. Intriguingly, *C. parvum*-infected animals developed digestive adenocarcinoma. In contrast, *C. muris*-infected mice did not develop neoplasia.

**Results**

Using their previously established model, the authors sought to identify metabolic pathways potentially involved in the development of *C. parvum*-induced ileo-caecal oncogenesis. They detected alterations in *Apc* and β-catenin expression in infected mice, implicating the Wnt pathway in *C. parvum*-induced neoplasia. Furthermore, immunohistochemical analysis revealed abnormal localization of β-catenin at a basolateral position, and transmission electron microscopy revealed dilation of intercellular spaces with development of lateral membrane extensions at the level of adherens junctions.

**Implications and future directions**

These data indicate that *C. parvum* is able to modulate host cytoskeleton activities and several host-cell biological processes. These effects could underlie the transformed phenotype of infected epithelial cells. There is increasing evidence to support an association between cryptosporidiosis and cancer. To better understand the implications for humans, further translational research is required to identify molecular and cellular mechanisms involved. The accumulation of clinical and experimental data in support of the association between the *C. parvum* and cancer, and the high exposure of humans and animals to this parasite, justifies the development of research on the topic, and provides incentive to use the animal model described herein.

Further analysis allowed us to show that different strains of *C. parvum*, which had been isolated from either animals or humans, induced digestive neoplasia in this rodent model (Certad et al., 2012; Certad et al., 2010a; Certad et al., 2010b; Certad et al., 2007), even in infections that had been induced with very low inoculum sizes (one to ten oocysts) (Benamrouz et al., 2012). In the same experimental model, the species *C. muris*, which has a gastric tropism (infecting mice and humans), did not induce that type of epithelial cell transformation, independently of the grade of immunosupression (Certad et al., 2010b).

Consistently, different evidence has shown direct or indirect association between cryptosporidiosis and cancer in different human populations – in a study among human immunodeficiency virus (HIV)-infected individuals, who are known to be highly susceptible to *Cryptosporidium* infections, the incidence of colorectal cancer was found to be higher than in the general population (Patel et al., 2008). Another study has reported that the risk of developing a colon carcinoma is significantly elevated among individuals with acquired immunodeficiency syndrome (AIDS) that present cryptosporidiosis (Shebl et al., 2012). A possible association between human cryptosporidiosis and liver cancer has been suggested in children with X-linked hyper-IgM syndrome (Tomizawa et al., 2004). Two epidemiological studies in Poland have reported a frequency of 18% and 12.6% of cryptosporidiosis in individuals with colorectal cancer of recent diagnosis before any immunosuppressive treatment (Sulżyc-Bielicka et al., 2012; Sulżyc-Bielicka et al., 2007).

Furthermore, it has been shown experimentally that *C. parvum* infection alters the gene expression profile of the host cell. These altered genes include those that are associated with apoptosis, such as BCL2 and the c-Myc proto-oncogene (Liu et al., 2009), proinflammatory signaling cascades and cytoskeletal dynamics (Deng et al., 2004). Nevertheless, even if we hypothesize that the acquired transformed phenotype of *Cryptosporidium*-infected epithelial cells is a consequence of modulation of cell signaling by the parasite, to our knowledge, no data about the mechanism of the induction of neoplasia by *C. parvum* is available.

The present work belongs to a series of experiments exploring the ability of *C. parvum* to induce neoplastic changes in the digestive epithelium of the mouse model. The aim of this study was to further characterize this animal model and to explore metabolic pathways potentially involved in the development of ileo-caecal neoplasia that is induced by *C. parvum* infection. To our knowledge, this is the first known parasitic protist able to induce epithelial invasive neoplasia in mammals (Benamrouz et al., 2012; Certad et al., 2012; Certad et al., 2010a; Certad et al., 2010b; Certad et al., 2007). Here, we searched for alterations in genes or proteins that are commonly involved in cell cycle, differentiation or cell migration, such as β-catenin and *Apc* (components of the Wnt signaling pathway), the *Kras* oncogene and p53 by molecular and immunohistochemical approaches.

## RESULTS

### Development of intraepithelial neoplasia and adenocarcinoma

The histopathological study showed the development of neoplasia of different grades of severity. Observed lesions varied from low-grade intraepithelial neoplasia (LGIEN) to well-differentiated invasive adenocarcinoma. A total of 27 dexamethasone-treated SCID mice were successfully infected with three different strains of *C. parvum* (IOWA, 20 mice; TUM1, four mice; and the IIaA15G2R1 strain isolated from a patient, three mice).

Upon histological examination of the ileo-caecal region, we observed LGIEN in two out of 19 mice that were euthanized after 40 days post-infection. The detected lesions were characterized by a slightly modified mucosal architecture, including irregular glands that were lined by cells that had slight atypias and that lacked, or contained minimal, mucin. In eight out of 19 animals, we discovered exophytic adenomas that showed an increasing architectural distortion, glandular crowding and major cellular atypias [high-grade intraepithelial neoplasia (HGIEN)]. In eight out of 19 mice, adenocarcinoma processes that invaded the submucosae through the muscularis mucosae were observed. In one out of 19 mice, we observed a well-differentiated adenocarcinoma that invaded the inner layer of the muscularis. In general, lesions showed a gradual progression from LGIEN to HGIEN and invasive well-differentiated adenocarcinomas that progressed into the lamina propria (intramucosal carcinoma), into the submucosa and through the muscularis mucosae into the subserosa. The severity of lesions increased steadily according to the delay post-infection. Those neoplastic lesions were accompanied by a diffuse inflammatory cell infiltrate, particularly in mice that had been infected with the TUM1 and IIaA15G2R1 strains. In summary, the incidence of ileo-caecal neoplasia was 100% (19 out of 19) in infected animals that were euthanized after 40 days post-infection.

### Apc labeling

**Fig. 1.**
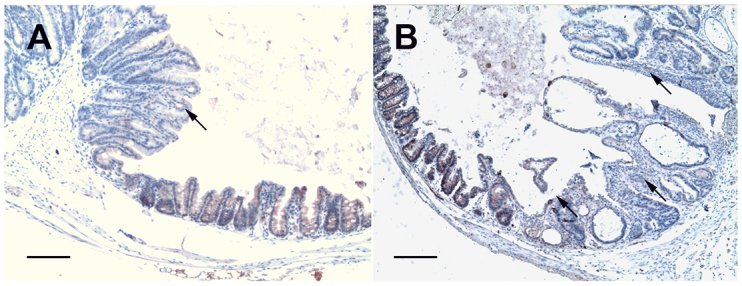
**Sections of ileo-caecal regions of dexamethasone-treated SCID mice immunostained for Apc.** (A) A polypoid-adenoma section from a mouse that was euthanized at 45 days post-infection showing a decrease of the intensity of cytoplasmic Apc labeling (arrow) after infection with *C. parvum*, whereas contiguous normal mouse tissue showed a staining pattern similar to that seen in a normal mucosa. (B) At 60 days post-infection, in a section of the ileo-caecal region taken from a mouse that had been infected with *C. parvum*, a decreased intensity of cytoplasmic Apc staining was observed in a polypoid-adenoma (arrow). Scale bars: 100 μm.

In the mucosa of ileo-caecal regions of uninfected control mice, Apc cytoplasmic immunoreactivity was detected in all animals. In all the infected animals that presented LGIEN, HGIEN or adenocarcinoma, a gradual decrease of the intensity of the labeling of cytoplasmic Apc after infection with diverse *C. parvum* strains was recorded in the lesions, whereas contiguous normal mouse tissue showed a staining pattern similar to that seen in normal tissue (Fig. 1). The decrease of Apc labeling was observed after 25 days post-infection and was found in lesions with either intraepithelial neoplasia or invasive adenocarcinoma (Table 1). The association between loss of Apc staining and a longer time post-infection was very significant (*P*<0.001). The incidence of this altered decrease of labeling was higher in animals that had higher amounts of parasite in tissues (*P*<0.001).

**Table 1.**
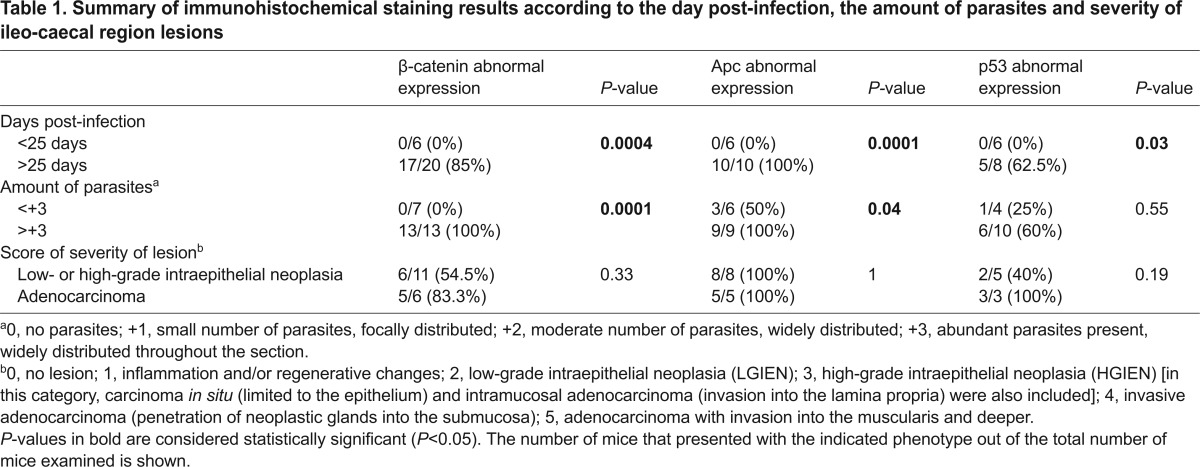
Summary of immunohistochemical staining results according to the day post-infection, the amount of parasites and severity of ileo-caecal region lesions

### β-catenin and E-cadherin labeling

**Fig. 2.**
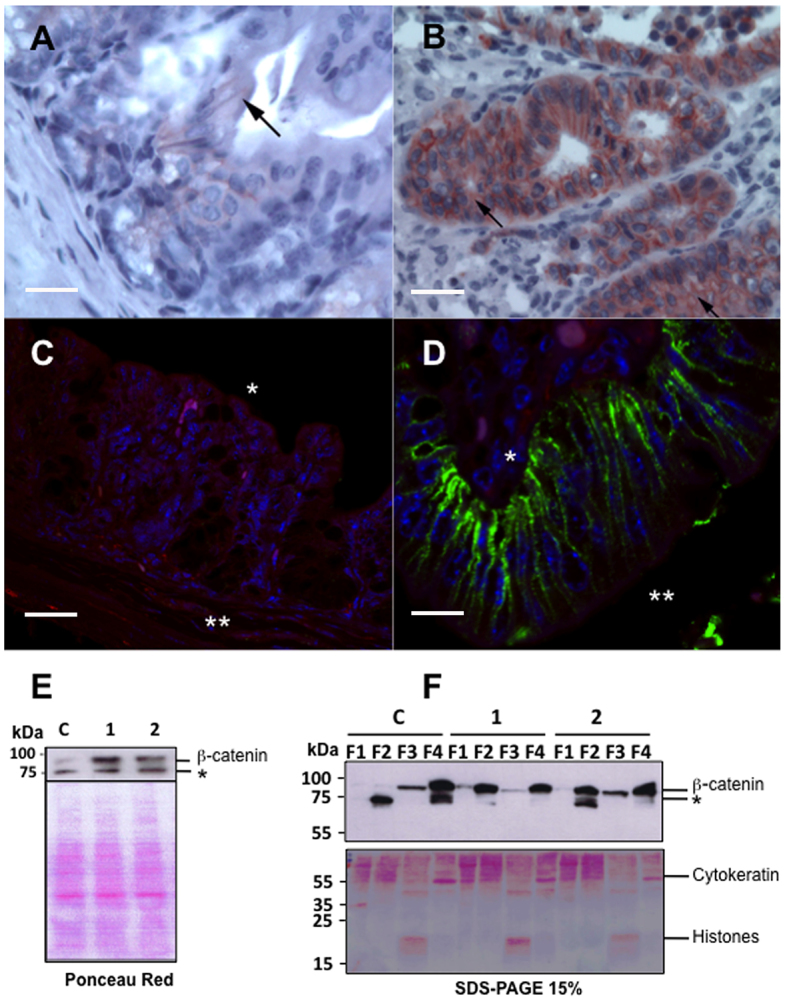
**Detection of β-catenin cellular localization by immunohistochemistry, immunofluorescence and western blot analysis.** (A) Section of normal non-neoplastic mucosa of the ileo-caecal region showed areas in which β-catenin could not be detected at the membrane, as well as areas that showed weak membranous expression of β-catenin (arrow). (B) Ileo-caecal epithelial cells from a *C. parvum*-infected mouse with invasive adenocarcinoma that was euthanized at 107 days post-infection show, after immunostaining for β-catenin, a persistent membranous expression, along with a prominent cytoplasmic staining (arrow). (C) Immunofluorescence microscopy analysis of a section of a normal non-neoplastic mucosa showed a lack of β-catenin expression using an antibody against the C-terminus of β-catenin and an Alexa-Fluor-488-coupled secondary antibody (green). * basal membrane, ** the lumen of glands. (D) Immunofluorescence microscopy analysis of ileo-caecal epithelial cells from a *C. parvum*-infected mouse with invasive adenocarcinoma, which was euthanized after 90 days post-infection showed membranous and juxtamembranous expression of β-catenin at a basolateral position by using an antibody against the C-terminus of β-catenin and an Alexa-Fluor-488-coupled secondary antibody (green). * basal membrane, ** lumen of glands. (E) Western blot analysis of the ileo-caecal region mucosa of mice (whole cell lysates) using an antibody against the N-terminus of β-catenin. β-catenin expression is higher in mice that had been infected with *C. parvum* (euthanized at 90 days post-infection, 1 and 2) when compared with a non-infected dexamethasone-treated control mouse (C), * unspecified bands. (F) Western blotting (top panel) using an antibody against the N-terminus of β-catenin after fractionation of epithelial cells showed traces of nuclear β-catenin (F3) in both the control (C) and *Cryptosporidium*-infected mice (1 and 2), the same degree of protein expression was observed. Increased β-catenin was observed in the F2 fraction (membrane and organelles) of *Cryptosporidium*-infected mice (1 and 2), * unspecified bands. F1, cytosol; F2, membranes and organelles; F3, nuclei; F4, cytoskeleton; C, control. Scale bars: 10 μm (A); 15 μm (B); 5 μm (C); 25 μm (D).

In the mucosa of the ileo-caecal regions of uninfected control mice, β-catenin labeling was weak and exclusively localized at the cell membrane. In six out of 11 (55%) mice that had been infected with *C. parvum* and exhibited LGIEN or HGIEN, and in five out of six (83%) mice that presented with adenocarcinoma, there was a progressive increase of the cytoplasmic labeling of β-catenin after 25 days post-infection, without loss of β-catenin membrane labeling. We did not observe nuclear β-catenin labeling, and β-catenin staining was more extensive in the cytoplasm of the cells with more severe lesions (Fig. 2A,B). The alteration of β-catenin expression was significantly associated with a longer time post-infection and with higher amounts of the parasite in tissues (*P*<0.001 and *P*<0.001, respectively); however, this alteration of β-catenin expression was not associated with the score of severity of the neoplastic lesions (Table 1).

In order to confirm our observations of the localization of β-catenin, more experiments were conducted. We performed immunofluorescence analysis using two different antibodies, one directed against the β-catenin C-terminus and the other against the N-terminus. We noticed a membranous and juxtamembranous localization of β-catenin in ileo-caecal tumor regions that had been obtained from infected animals presenting an invasive adenocarcinoma after 90 days post-infection; this accumulation of β-catenin was observed, mainly, at a basolateral position. The absence of β-catenin in the nucleus of ileo-caecal tumor regions after 90 days post-infection was confirmed (Fig. 2C,D).

Furthermore, western blot analysis was performed using the antibodies mentioned above. Firstly, on total cellular tumor lysates, an increase in β-catenin expression was observed in mice than had been infected with *C. parvum* and then euthanized 90 days post-infection when compared with non-infected dexamethasone-treated control mice (Fig. 2E). Secondly, after fractionation of epithelial cells that had been extracted from tumors, traces of nuclear β-catenin (in the F3 fraction) were observed in both control and *Cryptosporidium*-infected mice with the same degree of protein expression. In addition, an increased amount of β-catenin was found in the F2 fraction (membrane and organelles) of *Cryptosporidium*-infected mice when compared with control animals (Fig. 2F), and this difference was more apparent when using the antibody against the β-catenin N-terminus. Bands at 75 kDa were observed (Fig. 2E,F) in both control and infected mice. These are likely unspecified or cross-reactive bands; however, a proteolytic cleavage of β-catenin cannot be completely excluded.

Examination of E-cadherin revealed reduced staining of the protein in the host cell membrane after 45 days post-infection in animals that had been infected with *C. parvum* and that developed neoplastic lesions of the ileo-caecal region.

### Kras staining

We did not find any differences in the labeling of Kras in ileo-caecal epithelia, from both negative controls and *Cryptosporidium*-infected mice. At any time after infection, and at any stage of neoplasm progression, normal membrane staining of Kras was observed.

### p53 labeling

All animals failed to show nuclear expression of p53 in the adenomatous ileo-caecal region. At 25 days post-infection, cytosolic p53 labeling in the adenomatous cells was observed in five out of eight (63%) mice that had been infected with *C. parvum*. The alteration in the expression of p53 was significantly associated with the time post-infection (*P*=0.03) but not with the amount of the parasite in the tissues or the severity of the neoplastic lesions (Table 1).

### *Apc*, β-catenin and *Kras* mutations in tumors

In total, non-neoplastic intestinal tissue from non-infected SCID mice and five polypoid adenomas from four mice that had been infected with *C. parvum* were analyzed for three genes – *Apc*, β-catenin and *Kras* – using high-throughput sequencing. Analysis of the data showed that the same single nucleotide polymorphisms (SNPs) were detected in non-infected SCID mice and those that had been infected with *C. parvum*, as shown in Table 2. However, using a range of depth of 50× we did not find allelic variations in the regions of *Apc*, β-catenin and *Kras* that are considered the most likely to have mutations that are associated with colorectal cancer.

**Table 2.**
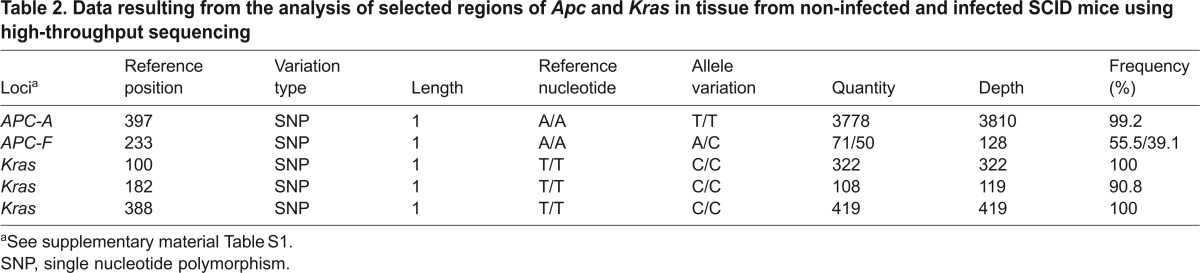
Data resulting from the analysis of selected regions of *Apc* and *Kras* in tissue from non-infected and infected SCID mice using high-throughput sequencing

### Electron microscopy

**Fig. 3.**
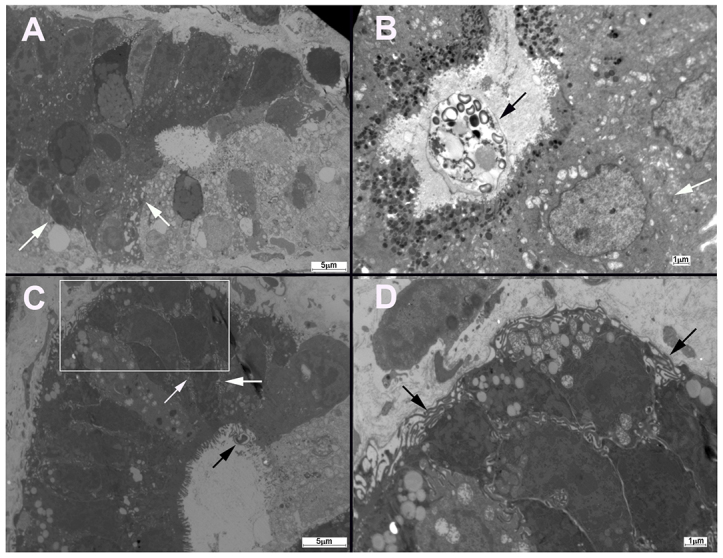
**Electron micrograph of ileo-caecal regions of dexamethasone-treated mice.** (A) Electron micrograph of a section of normal non-neoplastic mucosa that shows normal intercellular junctions (white arrows). (B) In SCID mice that had been infected with *C. muris* (black arrow), alterations in the ultrastructure of intercellular junctions (white arrow) of gastric epithelial cells were not found. (C) Dilation of intercellular spaces with extensive development of lateral membrane extensions (white arrows) was observed at the intercellular junctions of the ileo-caecal epithelia of mice infected with *C. parvum* (black arrow). (D) Enlarged image of the area indicate by the white box in C, which shows lateral membrane extensions (black arrows). Scale bars: 5 μm (A,C); 1 μm (B,D).

A dilation of intercellular spaces with extensive development of lateral membrane extensions was observed at the level of adherens junctions in the antropyloric and ileo-caecal neoplastic epithelia of mice that had been infected with *C. parvum* (Fig. 3). Mice that had been infected with *C. muris*, a species that has a gastric tropism but is not associated with digestive neoplasia, were analyzed to establish a comparison with those infected with *C. parvum*. Alterations in the ultrastructure of intercellular junctions of epithelial cells isolated from mice that had been infected with *C. muris* were not found.

## DISCUSSION

In the present study, using our rodent model of dexamethasone-treated SCID mice, we demonstrate for the first time that the Wnt signaling pathway is implicated during the progression of neoplasia that is induced by *C. parvum*, independently of the inoculated strain. This process seems to be atypical considering that, unlike colorectal adenocarcinomas, mutations in selected loci from crucial genes belonging to this signaling pathway (*Apc* and β-catenin) were not found, even if alterations in the expression of the immunostaining pattern were observed.

In our study, a gradual decrease of the intensity of cytoplasmic Apc labeling after infection with diverse *C. parvum* strains was recorded, associated with neoplastic lesions of the ileo-caecal region. *Apc* encodes a 2843-amino-acid cytoplasmic protein, one of the main functions of which is to facilitate the ubiquitylation and destruction of β-catenin in the Wnt signaling pathway (Kuraguchi et al., 2000). Nearly all of the tumor-associated somatic mutations in human *Apc* occur within the first 1500 codons, and approximately two-thirds of these mutations are confined to a mutation cluster region between codons 1286 and 1513 (Kuraguchi et al., 2000). When these mutations are present, a truncated Apc protein is unable to downregulate β-catenin. As a consequence, cytosolic levels of β-catenin are elevated, and the protein can enter into the nucleus where it can mediate transcriptional regulation. With the discovery of the association of the armadillo (ARM) repeats of Apc with proteins involved in cytoskeletal dynamics, another important functional role of Apc in cell morphology and migration through modifications of the actin cytoskeleton has become apparent (Buda and Pignatelli, 2004; Giles et al., 2003).

Using high-throughput sequencing, we examined in detail the regions of *Apc* that are considered most likely to have mutations in intestinal tumors in humans and mice between codons 789 and 1464 (Kuraguchi et al., 2000). It is well known that most human and rodent intestinal tumors contain mutations in these regions; however, in our study, mutations were undetectable by using high-performance methods. In a model of C57BL/6J Min/+ mice, it has been found that truncation of the Apc C-terminus was associated with reduced enterocyte migration and alteration of adherens junction proteins, and it has been hypothesized that these defects result from truncation of the Apc C-terminus, which contains microtubule-binding regions and putative sites for indirect binding to actin, and were related to changes in cytoskeletal function (Hughes et al., 2002). Therefore, it is possible that tumor cells induced by *C. parvum* might contain truncation mutations in *Apc* outside of the tested region.

In some cases, tumorigenesis is initiated by alterations in molecules other than Apc. β-catenin is a 92-kDa protein that, together with E-cadherin, plays a role in cell-cell adhesion and is involved in intracellular signaling. Colorectal adenomas and carcinomas show translocation of β-catenin from the cell membrane to the cytoplasm and/or nucleus, and this is considered as an early event in the development of colorectal neoplasia (Takahashi et al., 2000). In addition, mutation-mediated activation of β-catenin has been described in some instances of human colorectal cancer and this might bypass *Apc* mutations. In rats, cytoplasmic and/or nuclear translocation of β-catenin has been reported in azoxymethane-induced adenomas and carcinomas, and, in this model, impairment of Apc function is rare (Cooper et al., 2000). In our analysis, we found an abnormal juxtamembranous localization of β-catenin in 100% of mice that had intraepithelial neoplasia or adenocarcinoma, but we never observed nuclear expression of β-catenin, as confirmed by three different techniques (immunohistochemistry, immunofluorescence and western blot). Consistently, in transformed lymphocytes infected with Epstein-Barr virus, it has been reported that β-catenin was not found in the nucleus (Everly et al., 2004).

In addition to the alteration in β-catenin labeling, a reduced expression of the transmembrane protein E-cadherin was observed at the cell membrane of ileo-caecal epithelia after 45 days post-infection. E-cadherin forms the key functional component of adherens junctions of colonic epithelial cells. E-cadherin binds to catenins (α, β and γ) to form cytoskeletal complexes that are required for the maintenance of epithelial cell polarity and to preserve barrier function and intercellular adhesion (Buda and Pignatelli, 2004). Abnormalities in the cadherin-catenin complex, such as we observed, could result in reduced cell-cell adhesion and conversion to a migratory phenotype.

Nevertheless, we did not find oncogenic β-catenin mutations in tumor samples from mice that lack *Apc* mutations. Additionally, transcriptional silencing of *Apc* by promoter methylation has been suggested as an alternative to somatic mutation (Segditsas and Tomlinson, 2006). Another study has revealed the presence of concurrent methylation of groups of genes in hepatocellular carcinomas that are associated with infection with the hepatitis B or hepatitis C virus, suggesting that aberrant epigenetic changes that are associated with viral infection and exposure to environmental factors might activate events that promote the neoplastic transformation of hepatocytes (Lambert et al., 2011).

The role of p53 in human colitis-associated neoplasia has been studied by both immunohistochemistry and molecular techniques, and it has been reported that nuclear expression of, and the presence of mutations in, this gene can occur as early events, in contrast with non-colitic colorectal neoplasias (Cooper et al., 2000). In our study, we found accumulation of p53 in the cytoplasm of only those cells taken from infected mice after day 25 post-infection. Thus, the p53 signaling pathway seems to be involved in the process. These observations are consistent with another study that reported that *Theileria annulata* schizont leads to cytoplasmic sequestration of the majority of host-cell p53, resulting in the inhibition of p53-mediated apoptosis and the promotion of host-cell survival (Haller et al., 2010). Additionally, cytosolic accumulation of p53 in human colon cancer, caused by sequestration of the protein by the actin filaments, has been described (O’Brate and Giannakakou, 2003).

We did not examine the presence of possible mutations in p53, because of the fact that p53 genetic alterations in murine epithelial cells could be lower than those seen in humans. In fact, carcinogen-induced tumorigenesis in colorectal cancer in both mice and rats has resulted in conflicting reports regarding the molecular alterations and nuclear expression of p53 – some authors have reported no role, whereas others have reported a role for p53 (Cooper et al., 2000).

Along the known sequence of transformation from colonic adenoma to carcinoma, loss of *Apc* function is usually followed by oncogenic activation of *Kras* (Janssen et al., 2006). *Kras* encodes an intracellular signaling molecule, and its alteration results in constitutive activation of Ras and its downstream signaling pathways, playing an important role in cell death, differentiation and proliferation. Point mutations in *Kras* are among the most frequent genetic alterations in colorectal cancer in humans, and they have also been described in rodent models (Hu et al., 2009). After analysis of the proto-oncogene *Kras*, we did not find alterations in its expression or at genomic level. However, some SNPs, located in non-coding regions of the DNA of *Apc* and *Kras*, were detected in both neoplastic and non-neoplastic tissue samples. After analysis, it was found that these variations have not been previously reported in association with cancer. However, further exploration of this pathway could be performed in a future research work.

Based on the alterations we found in the expression of Apc and β-catenin, the Wnt pathway seems to be implicated in the induction of ileo-caecal adenocarcinoma by *C. parvum*. Furthermore, in neoplastic lesions, a stabilization of β-catenin, an abnormal accumulation at a basolateral position and a dilation of intercellular spaces with extensive development of lateral membrane extensions at the level of adherens junctions were observed. As described previously by other authors, these results might be interpreted as the recruitment of β-catenin to membrane ruffles (lamellipodia), which are implicated in the migration of transformed cells (Johnson et al., 2009; Odenwald et al., 2013).

Consistently, β-catenin was not observed in the nucleus of epithelial cells. Therefore, we hypothesized that there is also an activation of the non-canonical Wnt pathway, which involves Rho GTPase signaling without transactivation of the gene encoding β-catenin. For instance, it has been described that Rho GTPases and Wnt signaling are highly interconnected pathways that can influence hepatocarcinogenesis (Lechel and Rudolph, 2008). Furthermore, it is well known that *Cryptosporidium* infection induces cytoskeletal changes that modulate a localized actin reorganization and channel and transporter insertion, and it is likely that the signaling events that are initiated at the interface between host and pathogens induce whole-cell and, perhaps, tissue-level changes in the cytoskeletal architecture. These signaling axes include the phosphatidylinositol 3-kinase (PI3K), the guanine exchange factor Frabin-dependent activation of the small GTPase CDC42 and c-Src-dependent activation of cortactin (O’Hara and Chen, 2011).

In conclusion, the present results indicate that *C. parvum*, independently of the strain, is able to modulate host-cell cytoskeleton activities and several host-cell biological processes that could explain the transformed phenotype of infected epithelial cells. However, the mechanism of the specific cellular transformation induced by *Cryptosporidium* is still unclear. It is possible that a combination of several pathways is needed to transform infected cells. Then, we also have to consider the exploration of other signaling pathways in the future. It is of particular interest to investigate whether alterations in the Rho GTPase pathway contribute to colonic carcinogenesis.

How cells sense the pathogen and adjust their transcription and translation programs to their new life with a parasite remains an important issue (Cossart and Sansonetti, 2004). Furthermore, reports that suggest an association of cryptosporidiosis with cancer in humans, together with our experimental observations described herein, justify the development of research on the topic and support the use this original animal model to approach the subject.

## MATERIALS AND METHODS

### C. parvum oocysts

Oocysts of *C. parvum* IOWA and *C. muris* RN66 (purchased from Waterborne™, New Orleans, LA), *C. parvum* TUM1 (kindly provided by Donna Akiyoshi and Saul Tzipori, Tufts Cummings School of Veterinary Medicine, Boston, MA) and *C. parvum* IIaA15G2R1 (strain isolated from stools of an immunocompromised patient who developed fulminant cryptosporidiosis after near-drowning in Lille, France) were used. The stock solutions of oocysts were stored in a conservation medium [phosphate-buffered saline (PBS) or PBS with penicillin, streptomycin, gentamycin, amphotericin B and 0.01% Tween 20] at 4°C until use. Before inoculation, absence of other pathogens in the inoculum was confirmed by plating onto selective or non-selective culture media (Trypticase soy, Trypticase soy and blood, Hektoen, Tergitol 7 with triphenyltetrazolium chloride, Difco *Pseudomonas* Isolation Agar, Sabouraud). Oocyst viability was assessed by testing excystation (Certad et al., 2010b; Certad et al., 2007).

### Experimental hosts

Seven-week-old CB17-SCID mice were obtained from a colony bred at the Pasteur Institute of Lille (France) and regularly controlled for microbial (including *Helicobacter*) or parasitological pathogens. Animals were housed in groups in covered cages and maintained under aseptic conditions in an isolator with standard laboratory food and water *ad libitum*.

### Experimental design

SCID mice were administered with 4 mg/l of dexamethasone sodium phosphate (dexamethasone) (Merck, Lyon, France) through drinking water. Dexamethasone administration started 2 weeks before oral inoculation with *Cryptosporidium* oocysts and was maintained throughout the experiment. Dexamethasone-containing water was replaced three times a week.

For immunohistochemical and genetic studies, infective doses of 10^5^ oocysts per mouse of three different strains of *C. parvum* were prepared as described previously (Certad et al., 2012; Certad et al., 2010a; Certad et al., 2010b; Certad et al., 2007) and were inoculated by oral-gastric gavage to 27 animals (IOWA: 20 mice, TUM1: four mice and the IIaA15G2R1 human isolate: three mice). For ultrastructural studies, infective doses of 10^5^ oocysts of *C. muris* per animal were similarly prepared and inoculated. In order to localize β-catenin, seven additional animals were inoculated and followed for 90 days post-infection. In total, 12 negative control mice were inoculated only with PBS.

In order to determine parasite shedding, fecal specimens were collected and processed as described previously (Certad et al., 2010b). Periodically, or when signs of imminent death appeared, mice were euthanized by carbon dioxide inhalation. Assessment of the clinical condition of the mice was performed regularly to detect and then minimize suffering. Clinical signs that could constitute an endpoint included, but were not limited to, rapid or progressive weight loss, debilitating diarrhea, rough hair coat, hunched posture, lethargy or any condition interfering with daily activities (e.g. eating or drinking, ambulation or elimination).

Experiments were conducted in the animal facility of the Institut Pasteur de Lille (research accreditation number, A59107). Animal protocols were approved by the French regional ethical committee (approval number CEEA 112011).

### Histopathology and immunohistochemistry

Ileo-caecal regions were removed, fixed in 10% buffered formalin and processed using standard staining techniques (hematoxylin and eosin). Formalin-fixed and paraffin-embedded specimens were sectioned at a thickness of 5 μm, then all sections were deparaffinized, rehydrated through serial dilutions of alcohol and washed in PBS (pH 7.2). To assess and score the level of invasion of the neoplastic process, different techniques were used as described previously. The Volgens-Gomori stain (Bulckaen et al., 2008) was employed for assessing the gland basement membrane integrity. A monoclonal antibody against cytokeratin (AM071-5M, Biogenex, The Netherlands) was used to evaluate the invasion of epithelial cells into the lamina propria and into deeper organ layers. A monoclonal antibody against alpha smooth muscle actin (dilution 1:100) (M0851, Dako, Denmark) was used to stain muscle fibers in order to visualize muscularis mucosae disruption or penetration of the muscularis by neoplastic glands.

Other immunohistochemistry techniques were performed to explore the metabolic alterations that could be involved in the neoplastic process using antibodies against the following proteins: the C-terminus of β-catenin (clone H-102, sc-7199, Santa Cruz Biotechnology, Dallas, TX; rabbit polyclonal antibody used at a dilution of 1 in 125), Apc (sc-896, Santa Cruz Biotechnology, Dallas, TX; rabbit polyclonal antibody used at 1 in 100), wild-type and mutant p53 (ab4060, Abcam, Cambridge, UK; rabbit polyclonal antibody used at 1 in 50), E-Cadherin (mouse antibody against the human protein, clone NCH-38, used at 1 in 50) and Kras (12063-1-AP, Protein Tech, Chicago, IL; rabbit polyclonal antibody used at 1 in 100). Sections were immersed in pre-heated (95–100°C) citrate buffer (pH 6.0) for use with the antibodies against β-catenin, p53 and Kras, and in EDTA buffer (pH 8.0) for the antibody against Apc. For E-Cadherin staining, the sections were treated using an antigen retrieval technique and then rinsed in TBS buffer. Endogenous peroxidase activity was blocked by incubating with 3% hydrogen peroxide solution for 10 minutes. Then the samples were incubated with antibodies for 60 minutes at 25°C. Conventional biotin-streptavidin peroxidase was performed, and the slides were counterstained with hematoxylin. Normal tissues from non-infected mice or non-neoplastic regions of the same slide were used as controls for marker expression. Human colorectal and mammary adenocarcinomas were used, respectively, as positive controls for the antibodies against p53 and Kras. Sections were examined by two pathologists using a Leica DMRB microscope that was equipped with a Leica digital camera connected to an Imaging Research MCID analysis system (MCID software, Cambridge, UK).

Parasite load in digestive sections was scored on five selected fields at a magnification of ×400 as described previously (Certad et al., 2010a; Certad et al., 2010b). Histopathological lesions at different sites were scored as described previously (Certad et al., 2010a; Certad et al., 2010b) with slight modifications. Briefly, 0 – no lesion, 1 – inflammation and/or regenerative changes, 2 – low-grade intraepithelial neoplasia, 3 – high-grade intraepithelial neoplasia (in this category, carcinomas *in situ* and intramucosal adenocarcinomas were also included), 4 – adenocarcinoma (invading the submucosa), 5 – adenocarcinoma with invasion into the muscularis and deeper.

### Confocal immunofluorescence

Ileo-caecal regions were removed, fixed in 10% buffered formalin and embedded in paraffin. Sections that were 5-μm thick were placed on glass slides and deparaffinized using graded ethanol. This progressive rehydration was followed by an antigen retrieval step using citrate buffer pH 6.5 in a microwave oven for 15 minutes. After 30 minutes of blocking with PBSG buffer (PBS with 1.2% glycine, pH 7.3), the primary antibodies, diluted in PBSG, were applied for 1 hour at 37°C. After three 5-minute washes in the same buffer, the specimens were incubated in the secondary antibodies under the same conditions. After a final wash, DAPI was added for 10 minutes to stain the nuclei, and the sections were mounted in an anti-fading solution (Mowiol). Slides were visualized by using a Zeiss LSM780 confocal microscope: the 405-nm, 488-nm and 546-nm laser lines were used to excite DAPI, Alexa Fluor 488 and Alexa Fluor 546, respectively. The same instrument settings (laser power, scan speed) were used throughout the experiment in order to compare the labeling of the different specimens.

Primary antibodies against the C-terminus of β-catenin (clone H-102 described previously, used at a dilution of 1 in 125) and the N-terminus of β-catenin (clone E247, ab32572, Abcam, Cambridge, UK; rabbit monoclonal antibody used at 1 in 125) were used. Alexa-Fluor-488-conjugated anti-rabbit-IgG (1 in 500; Molecular Probes, Life Technologies, Carlsbad, CA) and Alexa-Fluor-546-conjugated anti-mouse-IgG (3 in 1000; Molecular Probes, Life Technologies, Carlsbad, CA) secondary antibodies were used.

### Fractionation of epithelial cells and western blot analysis

Visible tumors were collected from mice euthanized at day 90 post-infection and the epithelial cells were fractionated using the ProteoExtract^®^ Subcellular Proteome Extraction kit according to the manufacturer’s instructions (Calbiochem, Merck Millipore, Molsheim, France). For each fraction (F1 to F4), the protein concentration was measured using the micro BCA protein assay kit (Pierce, Fisher Scientific, Illkirch, France). Proteins (30 μg) were separated by using SDS-PAGE, and western blot analysis was carried out as described previously (Olivier-Van Stichelen et al., 2012) using two different antibodies against β-catenin (clones H-102 and E247 described previously, at a dilution of 1 in 5000).

### Ultrastructural study

Lesions, which had been identified macroscopically at the ileo-caecal region of mice infected with *C. parvum* and at the antropyloric region of mice that had been infected with *C. parvum* or *C. muris*, were fixed and embedded in EPON using glutaraldehyde 2.5% solution and then washed in PBS (0.1 M, pH 7.4) to optimize the preservation of cell structures (Aliouat et al., 1995; Palluault et al., 1992). Ultrathin sections were contrasted with uranyl acetate and lead citrate, and examined using a transmission electron microscope (LEO-906, Leica, Rueil-Malmaison, France).

### High-throughput sequencing

Five polypoid visible lesions and antropyloric regions from four mice that had been infected with *C. parvum* were dissected at day 90 post-infection. Additionally, normal tissue samples were obtained from non-infected control mice. Genomic DNA was prepared immediately using the NucleoSpin Tissue kit (Macherey-Nagel, Düren, Germany).

The regions of the *Apc* (Kuraguchi et al., 2000), β-catenin and *Kras* genes (Takahashi et al., 2000; Takahashi and Wakabayashi, 2004) that are considered most likely to have mutations associated with colorectal cancer in human and mouse were selected for primer design. Supplementary material Table S1 lists the corresponding primer pairs for 16 amplicons representing the *Apc*, β-catenin and *Kras* genes.

PCR protocols were performed by using genomic DNA that was amplified in 100-μl reactions containing 2 mM MgCl_2_, 250 nM primers, 250 μM dNTP and 5 U HotStart Taq (Qiagen). PCR reactions were performed in a 96-Well MasterCycler EP, Eppendorf PCR System. Cycle conditions included initial denaturation at 95°C for 5 minutes followed by 30 cycles of denaturation at 94°C for 20 seconds, annealing at 58°C for 15 seconds, extension at 72°C for 45 seconds and a final extension step lasting 4 minutes at 72°C.

After purification and quantification, the resulting amplicon products were pooled in an equimolar manner and barcode adaptor sequences were incorporated. The monitoring of the barcoded library was performed using a BioAnalyzer instrument (Agilent Technologies). Then, all the samples were pooled in an equimolar manner and, finally, the library was amplified by emPCR to obtain a sequencing template. The sequencing was performed using the Ion PGM 200 Sequencing Kit according to the manufacturer’s instructions (Life Technologies), and the data analysis was performed using CLC Genomics Workbench 5 modules.

### Statistical analysis

Fisher’s exact test (two-tailed) was used. Data analysis was performed by using the statistical software Graphpad. Significance was defined as *P*<0.05.

## Supplementary Material

Supplementary Material
