# Supplementary material for: Cryptosporidium parvum-induced ileo-caecal adenocarcinoma and Wnt signaling in a mouse model
Source: Dis Model Mech. 2014 Mar 20;7(6):693–700. doi: 10.1242/dmm.013292 (PMC4036476; doi:10.1242/dmm.013292)
Supplement: Supplementary Material [file supp_7_6_693__index.html]

Cryptosporidium parvum-induced ileo-caecal adenocarcinoma and Wnt signaling in a mouse model — Supplementary Material 

# *Cryptosporidium parvum*-induced ileo-caecal adenocarcinoma and Wnt signaling in a mouse model

## DMM013292 Supplementary Material

**Files in this Data Supplement:**

- **Supplementary Material**
