# Supplementary material for: Cryptosporidium parvum-induced ileo-caecal adenocarcinoma and Wnt signaling in a mouse model
Source: Dis Model Mech. 2014 Mar 20;7(6):693–700. doi: 10.1242/dmm.013292 (PMC4036476; doi:10.1242/dmm.013292)
Supplement: Supplementary Material [file supp_7.6.693_DMM013292.pdf]

Table S1. List of corresponding primer pairs for 16 amplicons representing *Apc*, β-catenin, and *Kras*

Name	Sens	ID GenBank, position	Sequence	Length	Tm(°C)
APC A	forward	NC_000084.6: 34312278-34312296	tcccggctcaagtctgcca	19	60,7
APC A	reverse	NC_000084.6: 34312690-34312709	gctatctgggctgcagtggt	20	59,4
APC B	forward	NC_000084.6:34312689-34312709	taccactgcagcccagatagc	21	58,7
APC B	reverse	NC_000084.6: 34313042-34313062	gggctaggtcagctggatact	21	57,8
APC C	forward	NC_000084.6: 34313041-34313062	cagtatccagctgacctagccc	22	58,8
APC C	reverse	NC_000084.6: 34313254-34313278	agacaggataactggtgttctggct	25	59,1
APC D	forward	NC_000084.6: 34313254-34313278	agccagaacaccagttatcctgtct	25	59,1
APC D	reverse	NC_000084.6: 34313734-34313756	gctgaacttggacgcagctgatt	23	60
APC E	forward	NC_000084.6: 34313734-34313756	aatcagctgcgtccaagttcagc	23	60
APC E	reverse	NC_000084.6: 34314176-34314195	gagcggagtctcctggacat	20	58,1
APC F	forward	NC_000084.6: 34314176-34314199	atgtccaggagactccgctcgtat	24	60,5
APC F	reverse	NC_000084.6: 34314466-34314485	accctctgcacggcagcatt	20	61,6
Kras	forward	NC_000072.6: 145247097-145247077	cctttgagagccattagctgc	21	56,2
Kras	reverse	NC_000072.6: 145246708-145246687	agcgttacctctatcgtagggt	22	56,2
beta-cat	forward	NC_000075.6: 120950465-120950490	cgtagatggcttcttcaggtagcatt	26	58,2
beta-cat	reverse	NC_000075.6: 120950787-120950807	gctgtcacacagccctgtcaa	21	59,7
